# Nutrition and allergic rhinitis: progress and perspectives on vitamin-mediated regulation

**DOI:** 10.3389/fnut.2026.1885368

**Published:** 2026-08-12

**Authors:** Jingwen Zhang, Yixi Xiao, Hengkang He, Xiong Zhang, Yuhong Lin, Peng Gao, Jing Luo, Yang Tian, Jianhui Zhang

**Affiliations:** 1 Department of Otolaryngology Head and Neck Surgery, The Affiliated Hospital of Southwest Medical University, Luzhou, China; 2 Southwest Medical University, Luzhou, China; 3 Department of Otolaryngology Head and Neck Surgery, The Third People’s Hospital of Chengdu, Chengdu, China

**Keywords:** allergic rhinitis, B vitamins, immunomodulation, vitamin A, vitamin C, vitamin D, vitamin E

## Abstract

Allergic rhinitis (AR) is a highly prevalent chronic inflammatory disease that imposes a substantial socioeconomic burden. Although conventional pharmacotherapy often provides symptomatic relief, recurrent exacerbations remain common in some patients. Micronutrients, particularly vitamins, have attracted increasing attention as potential immunomodulatory factors in AR. In this review, we identified relevant literature on vitamins, allergic airway inflammation, and immune-cell regulation from PubMed and Web of Science. Both direct evidence specific to allergic rhinitis and indirect evidence derived from asthma, broader allergic airway diseases, animal models, and *in vitro* cellular experiments were included, with a primary focus on distinguishing three categories of evidence: mechanistic rationale, observational associations, and clinical therapeutic efficacy. Vitamins A and D have been reported to promote regulatory T cell (Treg) differentiation and expansion while suppressing pathogenic T helper 17 (Th17) responses. Vitamin D may further stabilize mast cells, drive dendritic cells toward a tolerogenic phenotype, and enhance interleukin-10 (IL-10) production in B cells. Vitamin E and its isoforms exert anti-inflammatory effects by inhibiting mast cell activation, modulating group 2 innate lymphoid cell (ILC2) activity, and reducing Th2-type cytokine production. Extending beyond these classical immune-cell pathways, this review also discusses the potential role of vitamin-regulated microRNAs (miRNAs) in allergic inflammation. However, the strength of evidence varies substantially among vitamins. Current evidence for vitamins A, D, and E is supported by mechanistic findings, observational studies, and emerging clinical data, whereas evidence for B vitamins and vitamin C in AR remains relatively limited, inconsistent, or partly extrapolated from studies of asthma and other allergic airway diseases. Accordingly, the potential value of vitamin-based interventions should be understood within a personalized and context-dependent framework, shaped by baseline nutritional status, supplementation regimen, disease phenotype, concomitant therapy, and individual susceptibility. Further AR-specific mechanistic studies and large-scale, stratified, multicenter clinical trials are needed to clarify molecular mechanisms, validate clinical benefits, determine optimal supplementation strategies, and identify responsive populations, thereby providing a stronger evidence base for personalized nutritional approaches in the prevention and adjunctive management of AR.

## Introduction

Allergic diseases are a group of disorders caused by aberrant immune responses of the host immune system to otherwise harmless allergens. Over the past few decades, their prevalence has risen markedly worldwide, imposing a substantial socioeconomic burden on numerous countries. AR is estimated to affect approximately 29.4% of the global population (1). This condition is characterized by a chronic inflammatory process wherein allergens disrupt the nasal epithelial barrier and trigger a coordinated immune response involving multiple cell types. Dendritic cells (DCs) capture and present allergens to naïve CD4^+^ T cells, driving their differentiation into T helper 2 (Th2) cells. Th2 cells secrete interleukin-4 (IL-4), interleukin-5 (IL-5), and interleukin-13 (IL-13), which promote immunoglobulin E (IgE) class switching in B cells and subsequent IgE production by plasma cells (PCs). IgE binds to high-affinity immunoglobulin E receptors (FcεRI) on mast cells (MCs) and basophils; upon allergen re-exposure, cross-linking triggers degranulation and the release of histamine and other mediators. Eosinophils (Eos) infiltrate the nasal mucosa and release toxic granule proteins, sustaining late-phase inflammation. An imbalance between pro-inflammatory T helper 17 (Th17) cells and immunosuppressive regulatory T cells (Tregs), together with group 2 innate lymphoid cells (ILC2s) that serve as an additional source of Th2-type cytokines, further amplifies the allergic cascade. These events trigger and amplify oxidative stress, ultimately leading to nasal mucosal edema and excessive serous secretion. Symptoms including nasal congestion, rhinorrhea, postnasal drip, sneezing, and nasal pruritus markedly impair patients’ quality of life.

The development of allergic diseases is linked to multiple factors, including genetics, industrialization, sanitation, environmental exposure, and lifestyle. Notably, dietary changes are closely associated with the rising prevalence of allergic diseases. For example, in developing countries, rapid urbanization, dietary shifts, insufficient vegetable intake, and consumption of preserved foods are recognized as risk factors for chronic inflammatory and allergic disorders. Current conventional treatments for AR include allergen-specific immunotherapy, antihistamines, and intranasal corticosteroids. Nevertheless, these therapies mainly target symptom control, and their effectiveness may vary among patients, with recurrence still occurring in a subset of cases. Therefore, identifying safe adjunctive or complementary strategies that may improve symptom control and reduce recurrence remains an important research direction. Lifestyle and dietary patterns play a pivotal role in the development and progression of allergic diseases. In this context, the potential value of dietary bioactive compounds, particularly vitamins, in the prevention and adjuvant therapy of AR has attracted widespread attention in the scientific community (2). As essential micronutrients, vitamins not only maintain normal physiological functions but also exert immunomodulatory effects through multiple pathways (3–7). For vulnerable populations such as pregnant women and children, who require careful medication use, understanding the impacts of vitamins on AR progression, monitoring daily vitamin intake in patients with AR, and evaluating the potential of vitamin supplementation as a complementary strategy to conventional therapy may offer novel insights and directions for AR prevention and symptom control. However, current evidence remains heterogeneous, and the clinical value of vitamin supplementation should be interpreted cautiously according to vitamin type, baseline nutritional status, disease phenotype, and study design. Therefore, this review aims to critically summarize the available evidence, distinguish mechanistic plausibility from clinical efficacy, and identify the key limitations that should be addressed in future AR-specific studies.

### Vitamin A

Vitamin A (VitA) is a collective term for a group of fat-soluble organic compounds, mainly including retinol, retinal, and retinoic acid (RA). The human body cannot synthesize VitA *de novo* and must obtain it through dietary intake. Dietary VitA and its precursors are absorbed by intestinal epithelial cells, packaged into chylomicrons, and enter the circulation, eventually being stored in the liver, particularly in hepatic stellate cells, in the form of retinyl esters. When required by the body, retinyl esters are hydrolyzed into retinol, which binds to retinol-binding protein (RBP) and is released into the bloodstream. It is then transported to target tissues, where it is further metabolized into the active form RA. RA plays a critical role in immune regulation (8).

VitA and its active metabolites participate in the regulation of both innate and adaptive immunity, mediate immune cell differentiation and functional activation, maintain the structural and functional integrity of the airway epithelial barrier, and promote the normal development and differentiation of airway smooth muscle, thereby contributing to the suppression of allergic airway inflammation and airway hyperresponsiveness (4). In recent years, the prevalence of allergic diseases has continued to rise, and their occurrence is closely related to abnormal serum VitA levels induced by dietary changes. Multiple studies have shown that VitA levels in patients with allergic diseases are lower than those in healthy individuals, and serum VitA levels are negatively correlated with the risk of AR and other allergic diseases (4, 9–12). Within the intestinal mucosa, RA serves as a pivotal mediator linking dietary signals, the gut microbiota, and systemic immune regulation. CD103^+^ DCs in the lamina propria constitutively express retinaldehyde dehydrogenase (RALDH) and catalyze the biosynthesis of RA. Through retinoic acid receptors (RAR), RA induces the expression of the gut-homing receptors integrin α4*β*7 and CC chemokine receptor 9 (CCR9) on lymphocytes and synergizes with transforming growth factor-β (TGF-β) to promote the differentiation of forkhead box P3-positive (Foxp3^+^) inducible regulatory T cells (iTregs), thereby establishing oral tolerance to commensal bacteria and suppressing aberrant type 2 immune responses (8). Moreover, the interaction between VitA and the gut microbiota is bidirectional: certain commensals including *Prevotella* species harbor *brp/blh* family genes encoding putative *β*-carotene 15,15′ monooxygenases that can convert dietary provitamin A carotenoids into retinal, thereby potentially augmenting local RA production in the gut. Epidemiological evidence further supports the existence of this gut microbiota-VitA axis in AR. A cross-sectional study demonstrated a significant statistical interaction between retinol intake and gut *Prevotella* abundance: compared with individuals with low retinol intake and low *Prevotella* abundance, those with high retinol intake combined with high *Prevotella* abundance exhibited a 25-fold reduction in the adjusted odds of AR (11). This finding suggests that the protective association between dietary VitA and AR may partly depend on gut microbiota composition.

As a key molecule for maintaining immune homeostasis, RA plays a central role in regulating the balance between T helper 17 (Th17) cells and regulatory T cells (Tregs), thereby modulating helper T-cell immune responses (13). In animal studies, RA has been shown to exert anti-allergic effects in murine models of AR, primarily by inducing Tregs and suppressing Th2-type inflammation. RA can increase the mRNA levels of Foxp3, transforming growth factor-*β* (TGF-β), and interleukin-10 (IL-10) in mice, as well as the percentage of CD4^+^CD25^+^Foxp3^+^ T cells. It also reduces serum levels of IgE, IL-4, IL-5, IL-13, interleukin-17 (IL-17), interleukin-33 (IL-33), histamine, and leukotriene B4 (LTB4), and decreases goblet cell hyperplasia and excessive mucus secretion in the nasal mucosa (14, 15). In CD4^+^ T-cell culture systems, subsequent cell-based experiments confirmed that in an immune microenvironment containing TGF-*β*, RA significantly promotes the directed differentiation of CD4^+^ T cells into anti-inflammatory Tregs, synergizes with TGF-β to enhance the stable expression of Foxp3, and improves the phenotypic stability and immunosuppressive function of Tregs, thereby strengthening immune tolerance. RA can also directly act on CD4^+^ T cells to block interleukin-6 (IL-6)-mediated upregulation of retinoic acid-related orphan receptor γt (RORγt), inhibit Th17 cell polarization and the production of pro-inflammatory cytokines such as IL-17, effectively antagonize the pro-inflammatory effects of IL-6, and maintain Tregs/Th17 balance (16). Furthermore, RA helps maintain the phenotypic stability of CD4^+^ T cell subsets via microRNA (miRNA)-dependent mechanisms. In T-cell mechanistic studies, together with TGF-*β*, RA strongly induces miR-10a, which directly suppresses Bcl-6 and the corepressor Ncor2, thus restricting the differentiation of iTregs into follicular helper T cells (Tfh). When RA is present, miR-10a also inhibits Th17 polarization and diminishes IL-17A secretion through a T-bet (T-box expressed in T cells) pathway. These coordinated activities reinforce Treg lineage commitment and counteract Tfh and Th17 skewing (17).

Studies based on mast cell experimental models indicate that inhibition of the RAR can induce MC hyperactivation and degranulation, and inflammatory signaling pathways such as myeloid differentiation primary response protein 88 (MyD88)-IKK-nuclear factor-κB (NF-κB) and phosphatidylinositol 3-kinase (PI3K)-protein kinase B (Akt)-mTOR are involved in RA-mediated regulation of MC activation (18). In murine models of airway inflammation, the key functional isomer of RA, 9-cis-retinoic acid (9cRA), can increase the proportion of specific immunoglobulin A (IgA)-secreting B cells and serum IgA levels, while reducing serum IgE concentrations (19). Additionally, 9cRA promotes the differentiation of osteopontin (OPN)-producing DCs, which efficiently convert naïve T cells into Tregs and enhance immune tolerance (20).

Beyond its well-established role in adaptive T-cell regulation, RA also directly regulates innate lymphoid cells (ILCs), which are increasingly recognized as important contributors to mucosal type 2 inflammation in AR. In mouse intestinal ILC models and human ILC2 culture systems, VitA deficiency or RA exposure has been shown to alter ILC subset balance. Spencer et al. demonstrated that vitamin A deficiency markedly reduced intestinal group 3 innate lymphoid cells (ILC3s) and their production of interleukin-22 (IL-22) and IL-17, while ILC2s expanded significantly with elevated secretion of IL-13, IL-5, and IL-4. RA acted through RARα to intrinsically suppress ILC2 proliferation and IL-13 production in both mouse and human cells, establishing RA as a key suppressor of ILC2-driven type 2 responses (21). Consistent with these findings, studies in the human airway further support the regulatory role of RA in ILC2-mediated inflammation. Morita et al. showed that RA dose-dependently inhibited human ILC2 proliferation and IL-5/IL-13 secretion, and converted a subset of ILC2s into IL-10-producing regulatory ILCs (ILCregs). These ILCregs displayed a Treg-like phenotype (cytotoxic T-lymphocyte-associated antigen 4, CTLA-4^+^ CD25^+^) and suppressed the proliferation of both CD4^+^ T cells and ILC2s. Moreover, IL-13 induced the expression of retinaldehyde dehydrogenase 1 (RALDH1) in airway epithelial cells, indicating a negative feedback loop in which ILC2-derived IL-13 promotes epithelial RA synthesis, which in turn drives the conversion of ILC2s into ILCregs, ultimately limiting allergic airway inflammation (22, 23).

In addition to its broad immunomodulatory effects, RA may also influence allergen-specific immune responses. Regarding the widespread allergenic mold *Alternaria alternata (A. alternata)*, RA can bind to its major allergen Alt a 1, thereby masking IgE B-cell epitopes on the allergen surface, reducing IgE binding and MC degranulation. It also inhibits the activation of CD3^+^CD4^+^CRTh2^+^ Th2 cells, reduces pro-inflammatory cytokines such as IL-13, IL-6, and tumor necrosis factor-*α* (TNF-α), and increases tolerance-related factors such as IL-10, TGF-*β*, and IgA, thereby broadly suppressing Th2-mediated allergic inflammation and alleviating allergic responses *in vivo*. This provides a novel modification strategy for specific immunotherapy against *A. alternata* mold allergy (24). For special populations, existing clinical evidence from cohort studies indicates that maternal VitA intake during pregnancy is negatively correlated with the risk of AR in offspring (25). The mechanism may involve RA in utero regulating lymphoid tissue inducer (LTi) cells, thereby influencing the developmental size of secondary lymphoid organs and immune response efficiency in adult offspring (26). Moreover, serum VitA levels in children with AR are lower than those in healthy controls (27).

**Figure 1:**
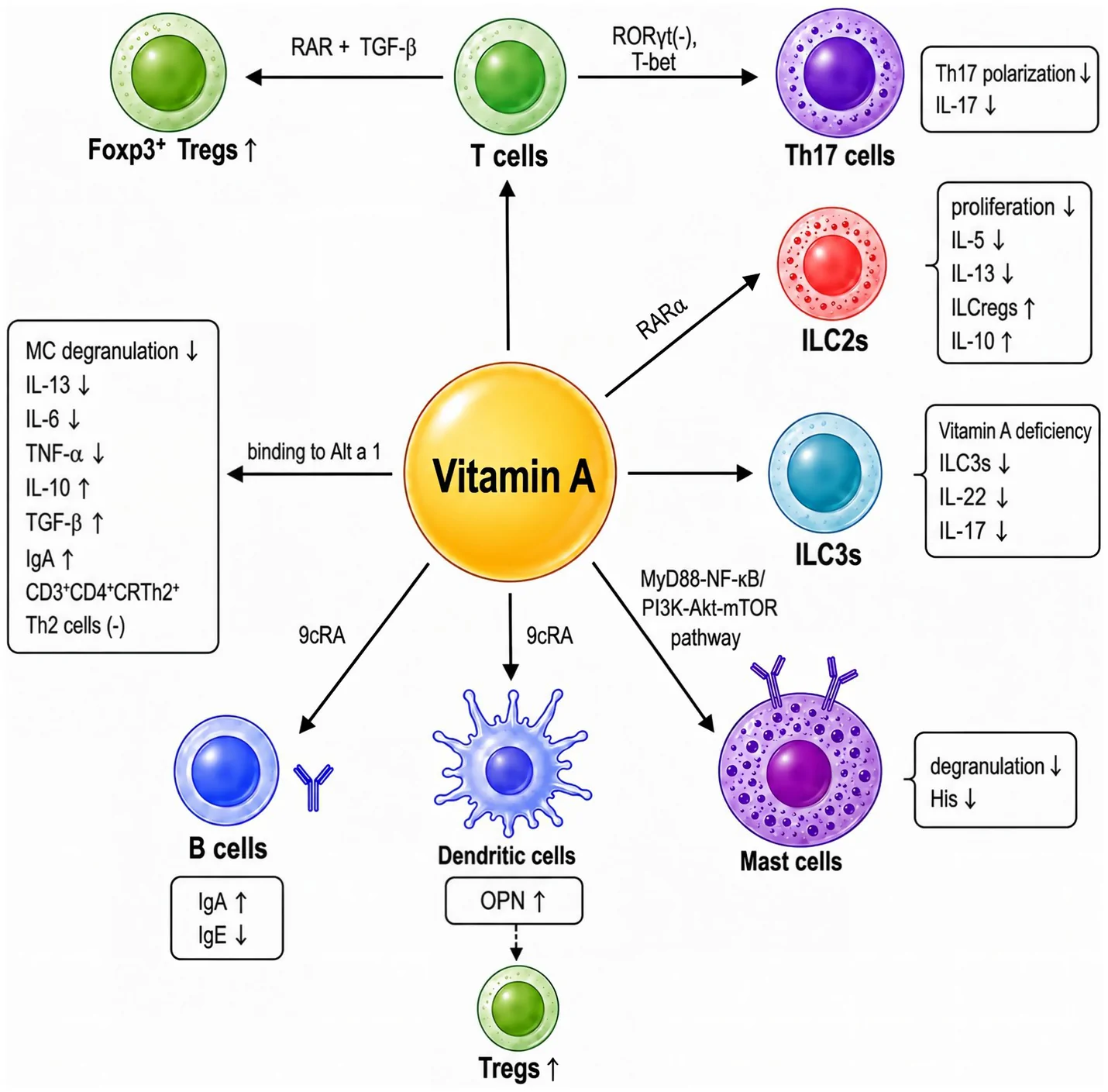
Schematic diagram of the immunoregulatory mechanisms of vitamin A in allergic rhinitis. In the diagram, vitamin A is placed at the center. Symbols are used consistently across all figures: → indicates regulatory direction, ↑ indicates upregulation or increase, ↓ indicates downregulation or decrease, and (−) indicates inhibition or suppression. Key signaling pathways or molecular targets mediating these effects are labeled on the arrows. The specific regulatory axes include: T cells: Through synergistic signaling of retinoic acid receptor (RAR) and transforming growth factor-*β* (TGF-β), vitamin A promotes the expansion of forkhead box P3-positive (Foxp3^+^) regulatory T cells (Tregs); simultaneously, via the retinoic acid-related orphan receptor *γ*t (RORγt) and T-box expressed in T cells (T-bet) pathways, it inhibits T helper 17 (Th17) cell polarization and reduces interleukin-17 (IL-17) secretion. Group 2 innate lymphoid cells (ILC2s): Via retinoic acid receptor α (RARα), vitamin A suppresses the proliferation of ILC2s and the production of interleukin-5 (IL-5) and interleukin-13 (IL-13), and induces their conversion into interleukin-10-positive (IL-10^+^) regulatory ILCs (ILCregs). Group 3 innate lymphoid cells (ILC3s): Vitamin A deficiency reduces ILC3 abundance and decreases the production of interleukin-22 (IL-22) and IL-17. Mast cells (MCs): Vitamin A inhibits MC degranulation and histamine (His) release through the myeloid differentiation primary response protein 88 (MyD88)-nuclear factor-κB (NF-κB) and phosphoinositide 3-kinase (PI3K)-protein kinase B (Akt)-mammalian target of rapamycin (mTOR) pathways. B cells: The vitamin A metabolite 9-cis-retinoic acid (9cRA) promotes immunoglobulin A (IgA) production and reduces immunoglobulin E (IgE) levels. Dendritic cells (DCs): 9cRA induces the differentiation of osteopontin (OPN)-producing DCs, and OPN in turn promotes Treg differentiation. Allergen interaction: After binding to the major allergen Alt a 1 of the mold *Alternaria alternata* (*A. alternata*), retinoic acid (RA) inhibits MC degranulation, downregulates interleukin-6 (IL-6), IL-13, and tumor necrosis factor-α (TNF-α), upregulates IL-10, TGF-β, and IgA, and suppresses the activity of cluster of differentiation 3 (CD3)^+^CD4^+^ chemoattractant receptor-homologous molecule expressed on Th2 cells (CRTh2)^+^ T helper 2 (Th2) cells.

VitA appears to regulate AR-related immunity through retinoic acid-dependent control of Treg induction, Th17 and ILC2 responses, mast cell activation, and IgA/IgE balance (Figure 1). This integrated pattern suggests that VitA may influence allergic inflammation not through a single immune target, but through coordinated regulation of adaptive immunity, innate lymphoid cells, effector-cell activation, and mucosal immune tolerance. Taken together, these findings highlight VitA as a promising candidate for nutritional intervention in AR. Future studies should clarify the relative contributions of these pathways across different patient populations and explore whether combined strategies targeting both VitA status and gut microbiota composition can achieve superior clinical outcomes.

### Vitamins with preliminary evidence: B vitamins and VitC

Compared with VitA, VitD, and VitE, the evidence supporting the roles of B vitamins and VitC in AR remains relatively limited and less clinically established. Therefore, these two vitamins are discussed together under a separate section to avoid overemphasizing their therapeutic relevance while preserving the comprehensive scope of this review. This structure reflects the current evidence hierarchy: B vitamins and VitC may be biologically relevant to immune regulation, epithelial barrier function, oxidative stress, and inflammatory control, but their AR-specific clinical evidence remains insufficient to support firm therapeutic conclusions.

### B vitamins

B vitamins are a group of water-soluble nutrients involved in diverse metabolic and immunological processes. Several B vitamins serve as key coenzymes in one-carbon metabolism, participate in epigenetic regulation such as DNA methylation, and modulate immune function, making them nutrients closely implicated in the pathogenesis of allergic diseases (28). Observational studies have demonstrated that maternal status of vitamin B2 (VitB2), vitamin B6 (VitB6), vitamin B9 (VitB9), and vitamin B12 (VitB12) during pregnancy is associated with the risk of atopic diseases in offspring (29, 30). At the mechanistic level, individual B vitamins have been shown to regulate inflammatory and immune responses through distinct pathways. Niacin, a major active form of vitamin B3 (VitB3), significantly suppresses the secretion of pro-inflammatory cytokines such as interleukin-1β (IL-1β), TNF-*α*, and IL-6 from macrophages under inflammatory stimuli through the GPR109A receptor (31). Biotin, also known as vitamin B7 (VitB7), modulates human monocyte-derived DCs via the AMP-activated protein kinase (AMPK) signaling pathway. Biotin deficiency suppresses AMPK activation in DCs, resulting in over-secretion of pro-inflammatory cytokines such as TNF-α, IL-1β, interleukin-23 (IL-23), interleukin-12 p40 (IL-12p40) and IL-6 upon lipopolysaccharide (LPS) stimulation. This in turn promotes the polarization of CD4^+^ T cells toward a pro-inflammatory Th1/Th17 phenotype, thereby enhancing inflammatory immune responses (32). Vitamin B6 (VitB6) modulates T lymphocyte proliferation and differentiation via the Janus kinase/signal transducer and activator of transcription (JAK/STAT) pathway, thereby maintaining immune balance. VitB6 deficiency suppresses T cell proliferation, lowers the CD4^+^/CD8^+^ ratio, and shifts the immune response toward a Th2-dominant profile, leading to decreased interleukin-2 (IL-2) secretion and elevated IL-4 levels with no significant change in interferon-*γ* (IFN-γ). It also downregulates the pathway inhibitor gene suppressor of cytokine signaling 1 (SOCS1) and upregulates T-bet transcription, ultimately impairing immune function (33).

Although the immunomodulatory effects of certain individual B vitamins have been preliminarily confirmed in basic mechanistic studies, direct evidence linking B vitamins to AR remains limited, and the available relevant evidence is mostly indirect and inferential. Most existing data are derived from studies on pregnancy-related atopic risk, macrophage cytokine production, dendritic cell function, or T-cell polarization, rather than AR-specific nasal mucosal inflammation or randomized supplementation trials in AR patients. Therefore, the current evidence does not justify a definitive conclusion that B-vitamin supplementation prevents or treats AR. Their specific molecular targets, regulatory mechanisms, and the preventive and therapeutic effects of individual B vitamins and diverse dosages in AR remain incompletely understood. No consensus has been reached regarding the specific roles and clinical value of B vitamins in AR. Future studies should first clarify whether B-vitamin deficiency exists in defined AR subgroups, then test correction of deficiency separately from pharmacological supplementation. Large-sample, multicenter, long-term follow-up clinical trials are warranted to validate their clinical efficacy. In addition, more targeted basic studies are needed to elucidate their mechanisms of action, so as to provide new theoretical bases and practical strategies for nutritional interventions in AR.

### Vitamin C

Vitamin C (VitC), also known as ascorbic acid (ASC), is an essential water-soluble micronutrient. It inhibits lipid peroxidation, thereby attenuating oxidative stress and alleviating the detrimental effects of reactive oxygen species (ROS). Furthermore, VitC elevates the levels of other antioxidants, including glutathione and *α*-tocopherol (α-T), while sustaining immune function and maintaining overall immune homeostasis. These properties suggest a potential beneficial role of VitC in the management of AR and other allergic diseases (34).

A long-term observational study reported that high-dose intravenous VitC alleviates allergy-related symptoms (35). In a subgroup of young children with AR, a South Korean cross-sectional study found that dietary VitC intake was inversely associated with AR symptom severity, whereas no significant differences were observed in serum total IgE levels or allergen sensitization (36). However, other observational studies have not established a clear association between VitC and atopic diseases (37). For instance, a cross-sectional study by Kompauer et al. (38) showed that plasma total carotenoid levels were inversely associated with AR prevalence, whereas plasma VitC levels were not correlated with allergic sensitization. A randomized, double-blind, placebo-controlled trial by Fortner et al. (39) found that neither high-dose nor low-dose VitC conferred any benefit against allergen-induced symptoms. These discrepant findings indicate that the clinical role of VitC in AR remains uncertain. At present, VitC should be regarded as a biologically plausible antioxidant candidate rather than an evidence-based therapeutic intervention for AR. Routine recommendation of VitC supplementation for AR symptom control is therefore premature without further well-designed trials.

In animal studies, existing evidence indicates that VitC exerts beneficial effects in asthma management. In an ovalbumin (OVA)-sensitized BALB/c mouse model, high-dose VitC shifted the Th1/Th2 balance toward a Th1-dominant profile during allergic airway inflammation (40). In addition, intranasal co-administration of choline chloride, VitC, and selenium showed significant therapeutic potential in a mouse model of allergic airway disease. This combined intervention effectively attenuated airway hyperresponsiveness, reduced inflammatory cell infiltration in lung tissue and bronchoalveolar lavage fluid, and downregulated the Th2 cytokines IL-4 and IL-5, as well as cockroach extract-specific IgE and IgG1 levels. Its core mechanisms involve suppressing NF-κB (p65) activation, decreasing oxidative stress markers, restoring glutathione peroxidase activity to rebalance airway redox homeostasis, upregulating IL-10 expression, and increasing the frequency of IL-10^+^ Foxp3^+^ Tregs, with superior anti-inflammatory and antioxidant efficacy compared with dexamethasone or *α*-lipoic acid monotherapy in this model. As a key exogenous antioxidant, VitC attenuates oxidative stress and type 2 airway inflammation by scavenging ROS and regulating NF-κB and IL-10 pathways. Although these findings support its potential as an adjuvant strategy for allergic airway inflammation, particularly asthma, their relevance to AR-specific nasal inflammation remains to be directly validated (34, 41).

In summary, although the antioxidant properties of VitC provide a biologically plausible rationale for its potential benefit in AR, the clinical evidence remains inconsistent and, compared with asthma, evidence supporting its efficacy in alleviating AR-specific symptoms remains limited. Most mechanistic evidence comes from asthma, generalized allergic airway inflammation, or oxidative-stress models, and its direct relevance to nasal mucosal inflammation in AR has not been sufficiently validated. Well-designed clinical trials and mechanistic studies specifically in AR populations are therefore needed to clarify its therapeutic value before any evidence-based recommendations can be formulated.

### Vitamin D

Vitamin D (VitD) is a fat-soluble micronutrient and one of the most commonly used dietary supplements. In recent years, its immunoregulatory role has attracted extensive attention. VitD critically sustains immune homeostasis by regulating Th1/Th2 immune balance.

However, the clinical evidence for VitD in AR should be interpreted cautiously. Although meta-analyses suggest an inverse association between serum VitD levels and AR risk, especially in children and males, these observational findings do not prove causality and may be affected by confounding factors such as sunlight exposure, lifestyle, baseline nutritional status, disease severity, and medication use (42–44). Interventional studies indicate that VitD supplementation may improve symptoms when used as an adjunct to antihistamines, intranasal corticosteroids, or allergen-specific immunotherapy, particularly in VitD-deficient or pediatric patients (45, 46). Nevertheless, these trials vary markedly in sample size, patient demographics, baseline VitD status, supplementation dosage and duration, concomitant therapy, and evaluated outcome measures. Thus, the observed clinical benefits may merely stem from the correction of underlying VitD insufficiency or synergistic effects with standard regimens, rather than a universal intrinsic anti-allergic property. VitD should therefore be regarded as a potential auxiliary therapy for selected AR subgroups. Among adult or mixed-age AR patients receiving intranasal corticosteroids or immunotherapy, adjunctive VitD has been reported to increase serum VitD levels and reduce TNSS compared with the same baseline therapy plus placebo; in pediatric AR patients undergoing sublingual immunotherapy (SLIT), supplementary VitD may further improve symptom control and immunological responses (47–52). Based on the above clinical evidence, VitD holds considerable potential as an adjuvant treatment for AR.

In specific populations, maternal VitD intake during pregnancy may exert long-term protective effects against certain allergic diseases in offspring. Multiple studies have confirmed that low maternal VitD levels during gestation are closely associated with an increased risk of atopic diseases in children (53–55). A prospective cohort study further indicated that sufficient VitD status in early pregnancy, particularly among women with normal pre-pregnancy body mass index (BMI), was associated with lower prenatal C-reactive protein (CRP) concentrations, which may partly explain the potential protective association between maternal VitD status and offspring atopic susceptibility (56). However, evidence from two meta-analyses and a prospective birth cohort study has not detected any beneficial effect of maternal or infant VitD supplementation on the development of atopic diseases in offspring (57–59). These inconsistencies suggest that VitD effects may be modulated by multiple factors, including baseline VitD levels, intervention timing, dosage, and genetic susceptibility.

VitD exerts its biological effects primarily through the vitamin D receptor (VDR), which is expressed in multiple immune cells (60). In Eos, the active metabolite 1,25-dihydroxyvitamin D3 (1,25(OH)₂D₃) binds to VDR and upregulates C-X-C chemokine receptor type 4 (CXCR4) expression, thereby counteracting IL-5-mediated inhibition of stromal cell-derived factor 1/C-X-C motif chemokine ligand 12 (SDF-1/CXCL12)-dependent chemotaxis and potentially redirecting Eos away from allergic inflammatory sites (61).

VitD also modulates innate lymphoid cell (ILC) plasticity. In human ILC2s, IL-1*β*, IL-23, and TGF-β can drive a shift toward IL-17-producing cells, characterized by ROR*γ*t upregulation and GATA-3 suppression. 1,25(OH)₂D₃ counteracts this transition by inhibiting RORγt transcription, thereby limiting IL-17A^+^ conversion and reducing IL-9 production (62). VDR-reporter mouse data further suggest that different ILC subsets may differ in their developmental dependence on VDR, although the functional significance of this distinction remains to be clarified (63).

In MC-focused experimental systems, 1,25(OH)₂D₃ upregulates VDR expression and promotes formation of a VDR-Lyn complex, which interferes with Lyn binding to the FcεRI β chain and MyD88. This suppresses downstream Syk phosphorylation and MAPK/NF-κB activation, thereby reducing histamine release and inflammatory mediator production. In parallel, VDR can directly repress TNF-*α* transcription by binding to the TNF-α promoter, reducing histone acetylation, and preventing the recruitment of RNA polymerase II and octamer-binding transcription factor 1 (OCT1). Moreover, MCs express 25-hydroxyvitamin D-1α-hydroxylase (CYP27B1), enabling local conversion of 25(OH)D₃ into active 1,25(OH)₂D₃; both metabolites can inhibit IgE-induced MC activation in a VDR-dependent manner (64, 65).

In activated neutrophil models, VitD attenuates neutrophil-mediated inflammation by suppressing activated neutrophil degranulation and reducing myeloperoxidase (MPO) release, thereby limiting MPO-associated tissue injury and mucosal barrier disruption. Mechanistically, VitD inhibits extracellular signal-regulated kinases (ERKs) and NF-κB signaling pathways, downregulates downstream pro-inflammatory mediators, including TNF-*α*, inducible nitric oxide synthase (iNOS), and cyclooxygenase-2 (COX-2), and decreases ROS production, thereby attenuating oxidative stress and the subsequent inflammatory cascade (66).

In human DC studies, VitD promotes a tolerogenic phenotype mainly through VDR-mediated metabolic reprogramming. Specifically, 1,25(OH)₂D₃ regulates 6-phosphofructo-2-kinase/fructose-2,6-bisphosphatase 4 (PFKFB4), increases fructose-2,6-bisphosphate (F-2,6-BP) production, activates phosphofructokinase-1 (PFK-1), and enhances glycolytic flux. This metabolic shift inhibits DC maturation, decreases co-stimulatory molecules such as major histocompatibility complex II (MHC II), CD40, CD80, and CD86, while increasing tolerogenic molecules and cytokines, including immunoglobulin-like transcript 3 (ILT-3), programmed death-ligand 1 (PD-L1), IL-10, and TGF-*β*. Consequently, VitD-induced tolerogenic DCs suppress pathogenic Th1/Th17 responses and promote Foxp3^+^ Treg differentiation, thereby strengthening peripheral immune tolerance and attenuating allergic inflammation (67, 68).

VitD modulates adaptive immunity by suppressing pathogenic Th17 responses and promoting Treg-mediated immune tolerance. Mechanistically, 1,25(OH)₂D₃ reduces C-C chemokine receptor type 6-positive (CCR6^+^) memory Th17 cells, downregulates CCR6 expression and pro-inflammatory cytokines, including IL-17A, IL-22, TNF-*α*, and IFN-*γ*, thereby limiting Th17-cell migration to inflammatory sites. It also shifts Th17 cells toward an anti-inflammatory phenotype by upregulating IL-10 and CTLA-4. In human CD4^+^ T-cell studies, 1,25(OH)₂D₃ promotes IL-10-producing and Foxp3^+^ regulatory T-cell differentiation, expands Foxp3^+^ Tregs, and restrains effector T-cell proliferation, thereby enhancing Treg stability and immunosuppressive function (69–71).

In human B-cell *in vitro* studies, VitD suppresses antibody-producing responses through VDR-dependent regulation. 1,25(OH)₂D₃ inhibits CD40/IL-21-driven B-cell differentiation, sustained proliferation of activated B cells, plasma cell (PC) and class-switched memory B-cell generation, and antibody secretion; local VitD activation via CYP27B1 may further reinforce these immunosuppressive effects (72, 73). 1,25(OH)₂D₃ associates with VDR and retinoid X receptor (RXR) to form a repressor complex that targets the microRNA-19a (miR-19a) promoter in B cells, suppressing its expression. This decrease in miR-19a releases IL-10 transcription from inhibition, restores IL-10 secretion, and contributes to counteracting the Th2-skewed response. Additionally, 1,25(OH)₂D₃ supplementation synergizes with allergen-specific immunotherapy (SIT) to further relieve AR symptoms (74). In addition, 1,25(OH)₂D₃ directly promotes IL-10 transcription via VDR binding to the IL-10 promoter and indirectly increases IL-10 secretion by upregulating transient receptor potential vanilloid 6 (TRPV6)-mediated calcium influx in activated human B cells (75).

**Figure 2:**
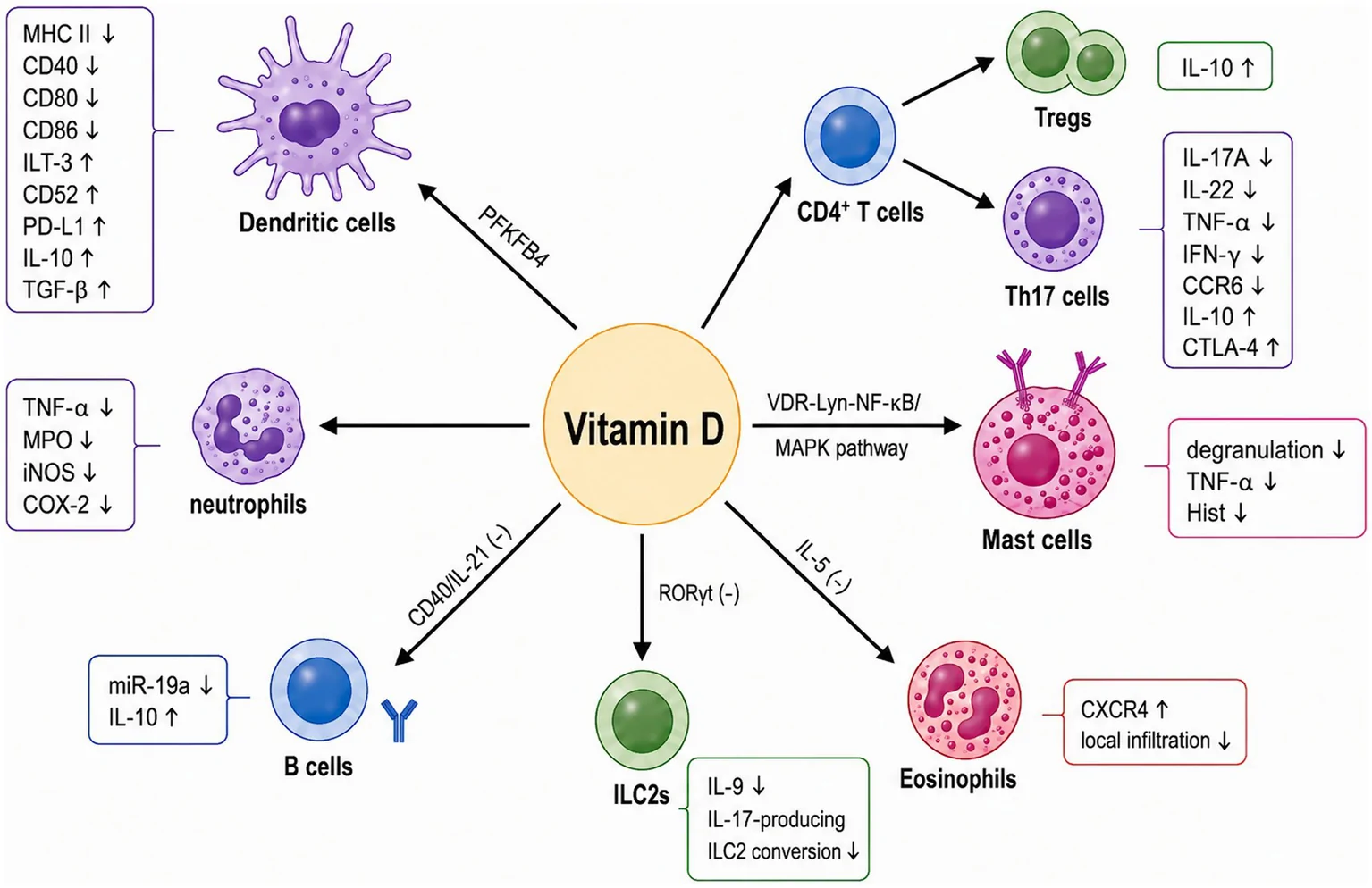
Schematic diagram of the immunoregulatory mechanisms of vitamin D in allergic rhinitis. In the diagram, vitamin D is placed at the center. Symbols are used consistently across all figures: → indicates regulatory direction, ↑ indicates upregulation or increase, ↓ indicates downregulation or decrease, and (−) indicates inhibition or suppression. Key signaling pathways or molecular targets mediating these effects are labeled on the arrows. The specific regulatory axes include: dendritic cells (DCs): Vitamin D remodels glucose metabolism by upregulating 6-phosphofructo-2-kinase/fructose-2,6-bisphosphatase 4 (PFKFB4), thereby inhibiting DC maturation. This results in downregulated expression of major histocompatibility complex class II (MHC II), cluster of differentiation 40 (CD40), CD80, and CD86, and upregulated expression of immunoglobulin-like transcript 3 (ILT-3), CD52, and programmed death-ligand 1 (PD-L1). It also promotes the secretion of interleukin-10 (IL-10) and transforming growth factor-β (TGF-β), driving DCs toward a tolerogenic phenotype. CD4^+^ T cells: Vitamin D promotes the differentiation of naïve CD4^+^ T cells into forkhead box P3 (Foxp3)^+^ regulatory T cells (Tregs) and enhances their IL-10 secretion; meanwhile, it suppresses T helper 17 (Th17) cells, downregulating the expression of interleukin-17A (IL-17A), interleukin-22 (IL-22), tumor necrosis factor-α (TNF-α), interferon-γ (IFN-γ), and C-C chemokine receptor type 6 (CCR6), while upregulating IL-10 and cytotoxic T-lymphocyte-associated protein 4 (CTLA-4), thus shifting Th17 cells toward an immunoregulatory phenotype. Mast cells (MCs): through the vitamin D receptor (VDR)-Lyn complex, vitamin D inhibits the nuclear factor-κB (NF-κB) and mitogen-activated protein kinase (MAPK) signaling pathways downstream of the high-affinity immunoglobulin E receptor (FcεRI), thereby reducing MC degranulation, histamine release, and TNF-α production, exerting a dual stabilizing effect. Eosinophils (Eos): by inhibiting interleukin-5 (IL-5) signaling, vitamin D upregulates C-X-C chemokine receptor type 4 (CXCR4) expression on the surface of Eos, redirecting their chemotaxis away from inflammatory sites toward non-inflamed tissues and reducing local infiltration. Group 2 innate lymphoid cells (ILC2s): by inhibiting retinoic acid-related orphan receptor γt (RORγt) transcription, vitamin D blocks the transdifferentiation of ILC2s into IL-17-producing cells, while also reducing interleukin-9 (IL-9) secretion. B cells: Vitamin D inhibits B cell differentiation and antibody production by blocking CD40/interleukin-21 (IL-21) signaling, and epigenetically modulates microRNA-19a (miR-19a) to relieve its suppression of IL-10, thereby enhancing IL-10 secretion by B cells. Neutrophils: Vitamin D suppresses neutrophil activation, reducing the expression of TNF-α, myeloperoxidase (MPO), inducible nitric oxide synthase (iNOS), and cyclooxygenase-2 (COX-2), as well as the production of reactive oxygen species (ROS), thereby alleviating oxidative stress and tissue damage.

Taken together, VitD is among the most extensively studied vitamins in AR and may act as a broad immune-homeostatic regulator. Lower serum VitD levels are associated with higher AR prevalence, and supplementation appears most relevant in VitD-deficient patients, pediatric populations, or as an adjunct to conventional therapy. Mechanistically, VitD coordinates a multicellular anti-inflammatory network by promoting PFKFB4-dependent tolerogenic DC programming, rebalancing Treg/Th17 responses, stabilizing MCs through the VDR–Lyn axis, regulating CXCR4-dependent Eos trafficking, limiting MPO/ROS-driven neutrophilic oxidative inflammation, modulating ILC2 plasticity, and enhancing B-cell IL-10 production through miR-19a- and TRPV6-related pathways (Figure 2). However, because much of this evidence comes from peripheral immune cells, in vitro systems, or lower-airway models rather than AR-specific nasal mucosal tissues, routine high-dose or universal VitD supplementation cannot yet be recommended; future studies should validate these mechanisms in nasal mucosa and define personalized adjunctive strategies.

### Vitamin E

As a crucial fat-soluble antioxidant, vitamin E (VitE) comprises four tocopherols (*α*-, *β*-, *γ*-, *δ*-tocopherols) and four tocotrienols (α-, β-, γ-, δ-tocotrienols). It can scavenge free radicals and maintain the stability of cell membrane structure and function. Moreover, it exerts broad immunomodulatory effects by regulating key signaling pathways and transcription factor activities, including inhibiting Th2-predominant immune responses, reducing inflammatory cytokine release, lowering IgE production, and suppressing MC activation and Eos infiltration, thereby alleviating allergic inflammation (3).

Animal models suggest that VitE supplementation may attenuate allergic airway inflammation by suppressing Th2 cytokines and oxidative stress, with evidence derived from both experimental AR and asthma-related models (76). Meanwhile, insufficient dietary intake of VitE is associated with olfactory and gustatory dysfunction, suggesting a possible protective role of VitE in sensory dysfunction that may accompany AR (77). Cross-sectional studies have found that VitE levels are lower in both children and pregnant women with AR compared with healthy individuals. Among pregnant women with AR, serum VitE levels are inversely correlated with TNSS, total IgE, IL-4, and IL-13 (78, 79). An intervention study has shown that VitE supplementation may relieve nasal symptoms in patients with seasonal allergic rhinitis (SAR) during pollen seasons, but has no significant effect on the duration of severe symptoms or the days of anti-allergic medication use (80). However, a cross-sectional study found no significant association between VitE intake and the severity of AR symptoms, nor with levels of cytokines and antibodies *in vivo* (36), and a randomized controlled trial reached a similar conclusion (81).

Mechanistic studies further support the anti-allergic potential of specific VitE isoforms, particularly α-T. In a murine asthma model, α-T transfer protein (TTP)-deficient mice subjected to OVA sensitization and challenge showed markedly reduced α-T levels. Following OVA sensitization and challenge, these mice exhibit significantly elevated expression of IL-5, TNF-α, and intercellular adhesion molecule-1 (ICAM-1) in lung tissue, as well as increased plasma IgE levels, accompanied by enhanced airway allergic inflammation. These findings indicate that α-T may suppress OVA-induced airway allergic responses by regulating the synthesis of inflammatory cytokines and immunoglobulins (82). In an allergic airway inflammation model focused on ILC2 biology, *α*-T downregulates the expression of scavenger receptor class B type 1 (SCARB1), and inhibits the liver kinase B1 (LKB1) signaling pathway, thereby promoting ferroptosis in ILC2s, and reduces the secretion of IL-5 and IL-13 (83). In a mouse model of AR, α-T can also inhibit the activation of the phosphatidylinositol 3-kinase/protein kinase B (PI3K/PKB) signaling pathway in MCs, thereby reducing the number of MCs and histamine release, decreasing eosinophil infiltration and the expression of Th2 inflammatory cytokines, further alleviating allergic inflammation in the nasal mucosa, and ultimately exerting an anti-allergic effect (84). In LPS-induced mature DCs *in vitro*, α-T can inhibit the activation of the NF-κB signaling pathway, thereby upregulating the expression of the anti-aging protein Klotho. In LPS-induced mature DCs, α-T treatment significantly reduces the expression of the costimulatory molecule CD86, inhibits the secretion of pro-inflammatory cytokines IL-12p70 and TNF-α, decreases ROS accumulation, and impairs CCL21-mediated cell migration. These results provide important mechanistic insights into how VitE may suppress early-stage allergic immune responses (85).

In addition to α-T, *γ*-tocopherol (γ-T) may also contribute to the anti-inflammatory effects of VitE, partly through its effects on reactive nitrogen species and eicosanoid pathways. In OVA-sensitized rat models of allergic airway inflammation, γ-T, an important active isoform of VitE, has been shown to exert anti-inflammatory effects by scavenging reactive nitrogen species (RNS) and alleviating nitrosative stress. Meanwhile, γ-T inhibits the activities of lipoxygenase (LOX) and cyclooxygenase (COX), thereby reducing the production of pro-inflammatory mediators including cysteinyl leukotrienes (Cys-LTs), leukotriene B4 (LTB4), monocyte chemoattractant protein-1 (MCP-1), and IL-6. γ-T significantly downregulates the expression of Th2 cytokines IL-5 and IL-13, markedly reduces eosinophil infiltration in the airways of OVA-sensitized rats, and specifically blocks ozone-induced enhancement of allergic airway inflammation, thus attenuating ozone-exacerbated mucous cell metaplasia and epithelial damage in the airways (86). In an experimentally induced allergic rhinitis and asthma model, γ-T suppresses allergen-induced expression of IL-4, IL-5, and IL-13 genes, reduces Eos infiltration in the entire respiratory tract including the nasal cavity, paranasal sinuses, lungs, and nasolacrimal ducts, alleviates mucous cell metaplasia and mucus hypersecretion in both upper and lower airways, and improves pathological changes in experimental AR and asthma (87). These findings suggest that γ-T may exert broader anti-allergic effects across the united airway; nevertheless, AR-specific clinical evidence remains insufficient, and the translational relevance of these experimental findings requires further confirmation.

In human mastocytoma HMC-1 cells, naturally occurring VitE inhibits cell proliferation through an antioxidant-independent mechanism. VitE selectively suppresses the PI3K/PKB/GSK signaling pathway and blocks PKB membrane translocation, thereby inhibiting HMC-1 cell proliferation. These findings provide a molecular mechanism for VitE as an intervention for MC-related allergic diseases. The inhibitory potency differs among isoforms, with the order δ-T > α-T = γ-T > β-T (88). In a rat inflammation model, γ-T exerts anti-inflammatory effects in vivo by inhibiting COX-2 activity, suppressing 5-LOX to decrease LTB4 levels, scavenging RNS, and downregulating TNF-α at high doses. Meanwhile, γ-T lowers inflammation-related lipid peroxidation marker 8-isoprostane and cell damage marker lactate dehydrogenase (LDH), and its metabolite γ-2,7,8-trimethyl-2-(β-carboxyethyl)-6-hydroxychroman (γ-CEHC) can also directly inhibit COX-2 to contribute to anti-inflammatory effects. In contrast, in this specific in vivo model, α-T failed to inhibit these pro-inflammatory pathways and mediators, suggesting that the anti-inflammatory effects of VitE may vary by isoforms, disease context, and outcome measures (89).

**Figure 3:**
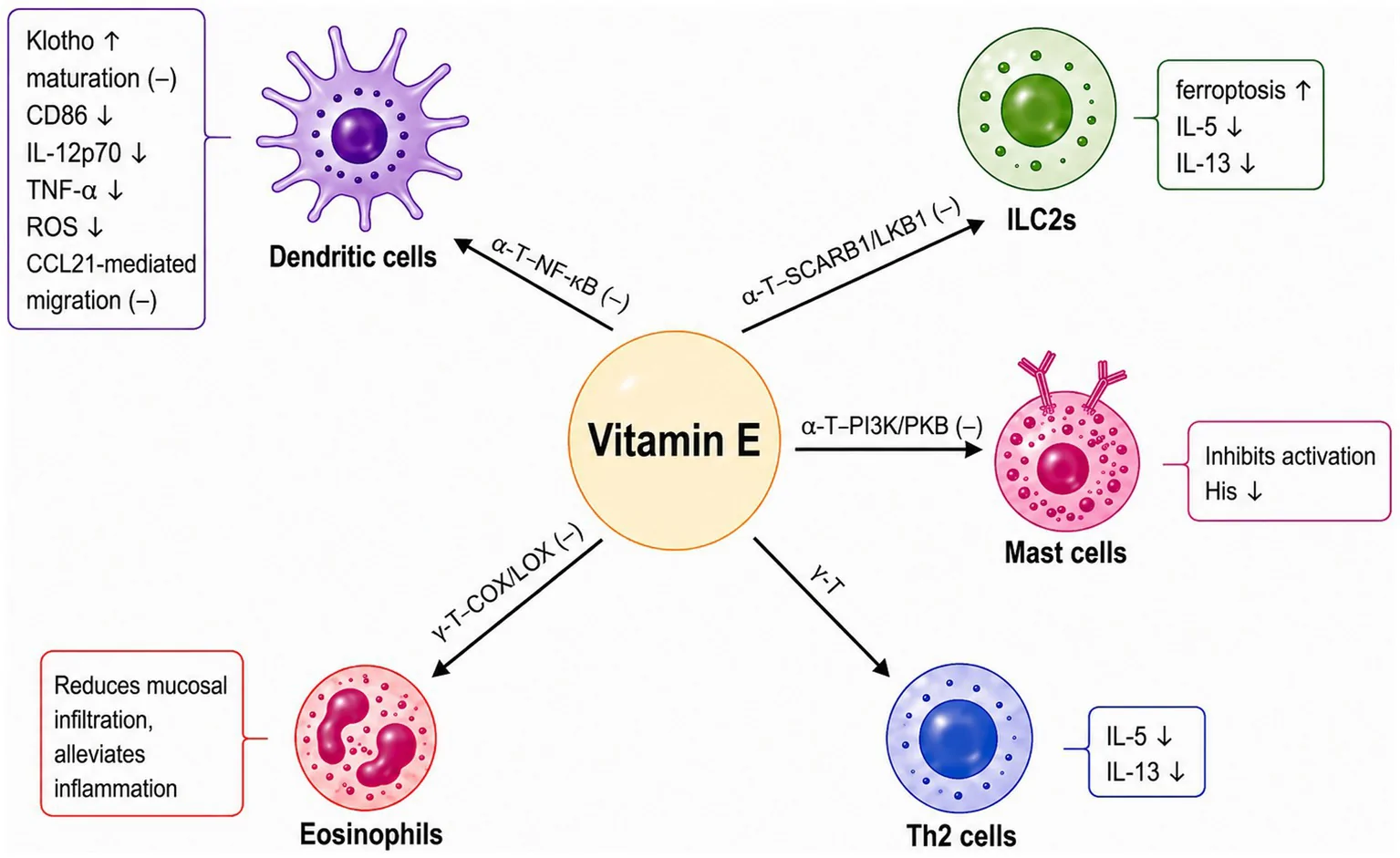
Immunoregulatory mechanisms of vitamin E in allergic rhinitis. Vitamin E is placed at the center. Symbols are used consistently across all figures: → indicates regulatory direction, ↑ indicates upregulation or increase, ↓ indicates downregulation or decrease, and (−) indicates inhibition or suppression. Key signaling pathways or molecular targets mediating these effects are labeled on the arrows. The specific isoforms mediating each effect, α-tocopherol (α-T) or γ-tocopherol (γ-T), are labeled on the corresponding arrows. The specific regulatory axes include: group 2 innate lymphoid cells (ILC2s): α-T downregulates scavenger receptor class B type 1 (SCARB1) and inhibits liver kinase B1 (LKB1) signaling, thereby inducing ferroptosis in ILC2s and reducing interleukin-5 (IL-5) and interleukin-13 (IL-13) secretion. Mast cells (MCs): α-T suppresses the phosphatidylinositol 3-kinase/protein kinase B (PI3K/PKB) signaling pathway, inhibiting mast cell activation and decreasing histamine (His) release. Dendritic cells (DCs): α-T inhibits the nuclear factor-κB (NF-κB) signaling pathway, upregulates the anti-aging protein Klotho, suppresses DC maturation, and consequently reduces the expression of the costimulatory molecule CD86, diminishes the secretion of pro-inflammatory cytokines interleukin-12p70 (IL-12p70) and tumor necrosis factor-α (TNF-α), decreases reactive oxygen species (ROS) accumulation, and impairs C-C chemokine ligand 21 (CCL21)-mediated migration. Eosinophils (Eos): through its anti-inflammatory properties, γ-T reduces eosinophil infiltration in the nasal mucosa and alleviates allergic inflammation. T helper 2 (Th2) cells: γ-T downregulates the expression of the Th2 cytokines IL-5 and IL-13.

VitE exerts protective effects in AR through multiple, isoform-dependent pathways. α-Tocopherol mainly targets MCs, DCs, and ILC2s by inhibiting PI3K/PKB-mediated MC activation and histamine release, attenuating DC maturation and inflammatory cytokine production, and facilitating LKB1-dependent ILC2 ferroptosis. In contrast, γ-tocopherol appears more closely associated with the suppression of eosinophilic and Th2-type inflammation, including reduced IL-4, IL-5, and IL-13 expression, IgE production, eosinophilic infiltration, and oxidative stress. This isoform-specific distinction is important for clinical translation, because total VitE intake or circulating VitE levels may not fully reflect the biological effects of individual tocopherol isoforms (Figure 3). Nonetheless, clinical findings remain limited and inconsistent; future studies should identify optimal active isoforms, dosing regimens, and the clinical value of VitE in AR.

## Discussion

This review summarizes the immunomodulatory properties of VitA, B vitamins, VitC, VitD, and VitE in AR from an immune cell-centered viewpoint. Although each vitamin displays unique regulatory actions on particular immune cell types, multiple vitamins frequently target the same cell populations. However, detailed insights into their synergistic, additive, or potentially opposing interactions remain insufficient. To better synthesize the translational relevance of the available evidence, the evidence hierarchy, dose ranges used in clinical or allergy-related studies, current clinical interpretation, and key knowledge gaps for each vitamin are summarized (Table 1). This raises an important conceptual issue: vitamins should not be viewed simply as isolated micronutrients with independent biological effects, but rather as components of an interconnected nutritional-immunological network. In such a network, the effect of one vitamin may depend on the status of others, the metabolic state of target cells, and the inflammatory context of the host. Therefore, the biological effect of vitamin supplementation in AR is likely to be nonlinear and context-dependent rather than uniformly beneficial.

**Table 1:** Evidence hierarchy, clinical dose ranges, and knowledge gaps for vitamin-mediated regulation in allergic rhinitis.

Vitamin	Level of evidence in AR	Dose ranges used in clinical trials	Synthesised clinical interpretation	Current knowledge gaps/future priorities	References
Vitamin A	Low to moderate. Strong mechanistic plausibility from RA-dependent immune regulation, animal AR models, and observational human studies; no AR-specific supplementation RCT was identified.	No validated AR therapeutic dose range. Existing human evidence is mainly based on serum levels, dietary/maternal intake, or non-AR atopy follow-up rather than AR treatment trials.	VitA may support mucosal immune tolerance by promoting Tregs, limiting Th2/Th17 and ILC2 responses, modulating MC activity, and shifting IgA/IgE balance. Clinical benefit remains associative rather than causal.	Clarify whether benefit depends on VitA deficiency, retinoid form, pregnancy/childhood exposure window, sex, and gut microbiota composition. AR-specific trials should also monitor safety because excessive VitA may be harmful.	(4, 8, 11–18, 21, 22, 25, 27)
B vitamins	Low. Evidence is mostly indirect, including pregnancy-related atopy associations and mechanistic studies on DCs, macrophages, and T-cell polarization; direct AR clinical efficacy data are lacking.	Not available for AR. No AR-specific supplementation regimen or dose range is supported by the cited evidence.	B vitamins may influence immune balance through one-carbon metabolism, AMPK-related DC regulation, JAK/STAT signaling, and cytokine production, but their relevance to nasal mucosal inflammation remains inferential.	Identify which B vitamin(s) matter most, distinguish correction of deficiency from pharmacological supplementation, and test AR-specific outcomes such as TNSS, quality of life, medication use, IgE, eosinophils, and cytokines.	(28–33)
Vitamin C	Low and inconsistent. Antioxidant rationale and animal airway data are available, but human AR/allergy evidence is limited and conflicting.	Oral ascorbic acid 2 g/day for 3 days, with dose–response testing at 0, 1, 2, and 4 g/day in a small allergen/histamine response study; IV vitamin C 7.5 g was used in an allergy-symptom observational subgroup. These are not validated AR treatment doses (35).	VitC may reduce oxidative stress and modulate NF-kB/IL-10 pathways, but RCT-level evidence has not confirmed a consistent clinical effect on allergen-induced nasal symptoms.	Conduct adequately powered AR-specific RCTs stratified by baseline VitC status, oxidative-stress burden, allergen phenotype, and route of administration. Standardized symptom, medication, and biomarker endpoints are needed.	(34–41)
Vitamin D	Moderate but heterogeneous. Supported by meta-analyses, several AR RCTs, and mechanistic studies, but effects vary by baseline 25(OH)D level, age, sex, and concomitant therapy.	AR trials used oral 800–1,600 IU/day or 1,000 IU/day with SLIT, and weekly high-dose regimens of 50,000–60,000 IU; reported treatment durations ranged from 4–8 weeks to 5 months. Dose should not be interpreted as a general recommendation (94).	VitD appears most promising as an adjunct in deficient patients, children, males, or patients receiving antihistamines or allergen immunotherapy. Evidence for pregnancy/infancy supplementation as long-term prevention remains inconclusive.	Future trials should predefine deficiency thresholds, standardize baseline therapy, evaluate sex/age and disease-severity subgroups, separate prevention from treatment, and validate mechanisms directly in nasal mucosa.	(5, 42–48, 51–59, 62, 64, 67, 69, 70, 72)
Vitamin E	Low to moderate and inconsistent. Human evidence includes observational studies and two AR intervention trials with divergent results; mechanistic and animal data suggest isoform-specific effects.	Seasonal AR trial: vitamin E 800 mg/day during pollen season. Perennial AR trial: vitamin E 400 IU/day for 4 weeks with loratadine-pseudoephedrine during the first 2 weeks. No validated AR dose range exists (81).	A positive seasonal trial suggested reduced nasal symptom scores, whereas a perennial AR trial found no significant effect on symptoms or specific IgE. Differences may reflect allergen seasonality, outcome measures, age, co-treatment, baseline antioxidant status, and tocopherol isoforms.	Distinguish alpha- and gamma-tocopherol effects, measure baseline VitE and oxidative/nitrosative stress, compare seasonal versus perennial AR phenotypes, and use unified endpoints including TNSS, medication use, IgE, eosinophils, IL-4, IL-5, IL-13, and quality of life.	(3, 36, 76–81, 83, 86–89)

AR, allergic rhinitis; DCs, dendritic cells; IgE, immunoglobulin E; IL, interleukin; ILC2, group 2 innate lymphoid cell; MCs, mast cells; NF-κB, nuclear factor-κB; RA, retinoic acid; RCT, randomized controlled trial; SAR, seasonal allergic rhinitis; SLIT, sublingual immunotherapy; TNSS, Total Nasal Symptom Score; Tregs, regulatory T cells; Vit, vitamin; 25(OH)D, 25-hydroxyvitamin D.

DCs serve as major targets for immune tolerance induction mediated by vitamins. VitD suppresses DC maturation by remodeling glycolytic metabolism (67, 68). α-T of VitE also restrains DC maturation via Klotho upregulation, manifested by lowered CD86 costimulatory molecule expression and decreased secretion of IL-12p70 and TNF-α (85). Conversely, insufficient biotin promotes DC polarization toward a pro-inflammatory profile through the AMPK pathway (32). Whether these vitamins cooperate or counteract each other within the same DC subset remains unclear. In addition, it is still unclear whether VitD acting via metabolic reprogramming and VitE relying on antioxidant and Klotho signals share common intracellular signaling hubs. One unresolved question is whether these vitamin-mediated effects converge on a common tolerogenic DC state or instead generate functionally distinct DC subsets with different capacities to induce Tregs, suppress Th2 responses, or regulate epithelial immunity. This distinction is important because a general reduction in DC maturation may not always translate into beneficial immune tolerance; excessive suppression of antigen presentation could theoretically impair protective mucosal immunity. Future investigations should apply single-cell multi-omics to delineate the molecular profiles of DCs exposed to various vitamin combinations and determine the optimal conditions required to establish immune tolerance.

The balance of T cell subsets constitutes a key regulatory outcome affected by multiple vitamins. Both RA and VitD facilitate Foxp3^+^ Treg formation and restrict Th17 cell differentiation (13, 16, 69). VitB6 deficiency triggers a Th2-biased immune pattern (33), whereas VitC and VitE primarily inhibit Th2-related cytokine secretion. Although these effects are relatively consistent *in vitro* and in animal models, the dose-dependent responses of different vitamins and the actual impacts of endogenous vitamin levels on T cell function in AR patients remain to be fully clarified. More importantly, studies exploring the combined effects of multiple vitamins on T cell subset polarization remain extremely limited. For example, RA acts through RAR and VitD signals via VDR to upregulate Foxp3 expression and support Treg development, yet whether they share common downstream transcriptional regulatory modules remains undetermined. Another important consideration is that restoration of immune balance may require different vitamin-dependent mechanisms at different disease stages. During early sensitization, vitamins that promote Treg development may help prevent the establishment of allergic immune memory. In contrast, in established AR, vitamins that suppress Th2 cytokine production, IgE responses, mast cell activation, or eosinophil recruitment may be more clinically relevant. This stage-specific interpretation may partly explain why preventive, observational, and therapeutic intervention studies often produce inconsistent results.

ILC2s have been identified as important effector populations in AR pathogenesis. VitA, VitD, and VitE exert inhibitory effects on ILC2s via distinct molecular mechanisms. RA represses ILC2 proliferation and drives their transition into a regulatory phenotype (21–23). VitD blocks the transdifferentiation of ILC2s into IL-17-secreting cells (62). VitE triggers ferroptosis in ILC2s (83). Further studies should specifically examine ILC2 subsets in nasal mucosal samples from AR patients, rather than relying solely on lower-airway immune models. This point is particularly important because AR and asthma share type 2 inflammatory features but differ in tissue architecture, epithelial exposure, microbial environment, and local immune cell composition. A major limitation of current mechanistic evidence is that many findings are derived from asthma models, allergic lower-airway inflammation models, peripheral blood immune cells, or in vitro systems rather than AR-specific nasal mucosal tissues. Although AR and asthma share several type 2 inflammatory features, including epithelial barrier dysfunction, IgE-mediated mast cell activation, eosinophilic inflammation, and ILC2 activation, they differ in tissue architecture, local allergen exposure, microbial environment, and immune-cell composition. Therefore, findings from asthma or generalized allergic airway disease should be interpreted as indirect mechanistic support rather than direct evidence of therapeutic efficacy in AR. Future studies should prioritize nasal epithelial cells, nasal lavage fluid, inferior turbinate tissues, and nasal mucosal immune-cell profiling from AR patients to validate these mechanisms in disease-relevant tissues.

MCs and Eos directly mediate the clinical symptoms of AR. VitD exerts a well-defined MC stabilizing function by binding VDR to Lyn and inhibiting the NF-κB pathway (64, 65). VitD also promotes the redistribution of Eos from inflamed regions to non-inflamed tissues (61). α-T attenuates MC activation by suppressing the PI3K/PKB pathway (84), while γ-T lessens eosinophil infiltration (86, 87). RA from VitA limits excessive MC degranulation through RAR signaling (18). VitC is capable of regenerating oxidized VitE, but whether such antioxidant crosstalk enhances biological functions in AR remains experimentally unproven. Future studies should establish ex vivo functional assays using human MCs and Eos treated with single or combined vitamins to evaluate multi-dimensional outcomes including degranulation, chemokine production, and apoptosis. From a clinical perspective, these effector-cell mechanisms may be more closely related to symptom relief than to disease modification. Therefore, future trials should not rely solely on global symptom scores, but should also incorporate mechanistic endpoints such as nasal eosinophil counts, local histamine levels, epithelial cytokines, and changes in nasal lavage inflammatory mediators. Such endpoints may help determine whether vitamin supplementation truly modifies allergic inflammation or merely reflects general nutritional improvement.

In terms of B cells and IgE production, both VitA and VitD suppress IgE class switching and plasma cell differentiation but through distinct mechanisms. VitA promotes IgA synthesis while inhibiting IgE formation (19), whereas VitD globally restrains B cell activation and enhances IL-10 secretion (72, 73, 75). However, no existing study has explored receptor crosstalk or synergistic actions between these two vitamins in B cells. Additionally, B vitamins participate in one-carbon metabolism and DNA methylation, which may exert long-lasting influences on B cell epigenetic regulation. For instance, maternal folate levels during pregnancy are associated with offspring atopic disease risk (28), but related data in the field of AR remain largely lacking. This suggests that vitamins may influence AR not only through short-term immune regulation but also through longer-term immune programming. Maternal vitamin status, early-life nutrition, and epigenetic imprinting may shape allergic susceptibility before clinical symptoms appear. However, this possibility also complicates interpretation: vitamin exposure during pregnancy or early childhood may have effects that differ from supplementation after AR has already developed.

A major limitation of current research is the discrepancy between mechanistic plausibility and clinical certainty. While studies show that vitamins modulate AR-relevant immune cells, including DCs, Tregs, Th17 cells, ILC2s, MCs, Eos, and B cells, these findings do not necessarily translate into clinical efficacy. Mechanistic data are derived largely from asthma models, generalized allergic airway inflammation models, peripheral immune cells, or in vitro systems, with limited direct evidence from nasal epithelium, nasal lavage fluid, inferior turbinate tissue, or AR-specific mucosal immunity. Observational associations between serum vitamin status and AR risk may be confounded by diet, sunlight exposure, lifestyle, disease severity, socioeconomic factors, and medication use. Clinical trials also exhibit substantial heterogeneity in dosage, intervention duration, baseline vitamin status, demographics, disease phenotype, outcome measures, and background therapy, which may partly explain the inconsistent efficacy findings. Collectively, current evidence supports vitamins as modulators of AR-related immune responses, but does not yet establish them as independent or universal therapeutic agents for AR. To further clarify the disease source and translational relevance of the cited evidence, studies discussed in this review are classified according to whether they provide direct AR evidence, extrapolated evidence from asthma or allergic airway inflammation, or non-AR mechanistic evidence (Table 2).

**Table 2:** Disease-source-oriented distinction of evidence for vitamins in allergic rhinitis, asthma, and other allergic diseases.

Evidence category	Study object/disease	Vitamin(s)	Source references	Evidence type	Main mechanism	Main findings/impact and relevance to AR
AR direct evidence	Allergic rhinitis; diet and gut microbiota	VitA	(11)	Cross-sectional/epidemiological study	Interaction between retinol intake and gut Prevotella abundance suggests that a VitA–gut microbiota–immune tolerance axis may participate in AR regulation.	High retinol intake combined with high Prevotella abundance was associated with lower AR risk. This supports an association between VitA and reduced AR risk, but does not prove a therapeutic effect of supplementation.
	AR animal models; some studies also involved asthma	VitA/RA	(15)	Animal experiment/mechanistic study	RA promotes Foxp3^+^ Treg differentiation, suppresses Th2/Th17 responses, and reduces IgE, IL-4, IL-5, IL-13, IL-17, IL-33, histamine, and LTB4.	RA alleviates nasal mucosal inflammation, goblet cell hyperplasia, and mucus secretion. This supports AR-related mechanisms; studies involving asthma should be described within a united-airway inflammation context rather than as direct AR clinical efficacy.
	Maternal vitamin intake and offspring AR risk	VitA	(25)	Birth cohort/observational study	Maternal VitA status may affect lymphoid tissue development and immune programming through fetal RA signaling.	Maternal VitA intake was associated with reduced offspring AR risk. This is suitable as direct observational evidence linking early nutritional exposure with AR risk.
	Serum fat-soluble vitamin levels in children with AR	VitA, VitD, VitE	(27)	Clinical observation/propensity-score matching	Reflects the relationship between fat-soluble vitamin status and AR-related immune-inflammatory phenotypes.	Children with AR had lower levels of relevant vitamins than healthy controls. VitK is not listed separately because the manuscript does not discuss it as an independent mechanism or treatment for AR.
	Children with AR; dietary antioxidant nutrients	VitC, VitE	(36)	Cross-sectional study	VitC and VitE may influence ROS-related inflammation through antioxidant pathways, but the evidence is inconsistent.	Dietary VitC intake was inversely associated with AR symptom severity. The association of VitE with AR severity, cytokines, or antibodies was unstable. This is AR observational evidence, not proof of supplementation efficacy.
	Adult AR and allergic sensitization	VitC	(38)	Cross-sectional study	Assessed the relationship of plasma antioxidant nutrients with AR and allergic sensitization.	Plasma VitC showed no clear association with allergic sensitization. The original study reported signals for carotenoids, but carotenoids are not systematically discussed in this manuscript and are therefore not included as independent vitamin entries.
AR-related direct evidence	Nasal/skin histamine and allergen-induced responses	VitC	(39)	Randomized, double-blind, placebo-controlled trial	Tested whether VitC reduces induced responses through antioxidant or antihistamine-related effects.	Neither high-dose nor low-dose VitC improved allergen-induced symptoms. This should be described as a negative RCT on nasal/skin provocation responses, not as positive modern AR treatment evidence.
AR direct evidence	Children/general population with AR; serum VitD levels	VitD	(42–44)	Systematic review/meta-analysis	VitD regulates Th1/Th2 balance, Tregs, DCs, B cells, mast cells, and eosinophils through VDR-dependent pathways.	Lower serum VitD levels were associated with increased AR risk in children, and some studies suggested sex-related differences. VitD has one of the more complete evidence chains among the vitamins discussed.
	Routine AR treatment combined with VitD	VitD	(45, 46)	Clinical intervention/RCT	VitD combined with antihistamines may reduce IL-4 and peripheral eosinophils and improve Th2 inflammation.	Symptoms improved in VitD-deficient AR patients. This should be presented as adjunctive-treatment evidence and not extended to routine high-dose supplementation for all AR patients.
	AR patients receiving intranasal corticosteroids or SLIT combined with VitD	VitD	(47, 48, 51, 52)	Clinical trial/adjunctive-treatment study	May promote immune tolerance, enhance Treg/Breg/IL-10-related responses, and improve immunotherapy efficacy.	TNSS and serum VitD levels improved; VitD combined with SLIT in children may enhance clinical and immunological responses. The evidence should be framed as add-on use with standard therapy.
	Mouse AR model	VitE/α-tocopherol	(84)	Animal experiment/mechanistic study	α-Tocopherol suppresses mast-cell PI3K/PKB signaling, reducing mast-cell activation, histamine release, eosinophil infiltration, and Th2 cytokine expression.	Attenuates allergic inflammation in the nasal mucosa. This is direct mechanistic evidence from an AR animal model, but not clinical efficacy evidence.
	Patients with seasonal AR and perennial AR	VitE	(80, 81)	Clinical intervention/RCT	Tested whether antioxidant and anti-inflammatory effects reduce nasal symptoms and IgE-related responses.	The seasonal AR study showed partial relief of nasal symptoms but did not significantly reduce the duration of severe symptoms or medication-use days; the perennial AR study showed no clear benefit. These findings indicate inconsistent clinical effects of VitE.
	Children with AR; pregnant women with AR	VitE	(78, 79)	Clinical observational study	VitE may influence AR by reducing oxidative stress, Th2 cytokines, and IgE-related responses.	Serum VitE levels were lower in children and pregnant women with AR. In pregnant women with AR, VitE was inversely associated with TNSS, IgE, IL-4, and IL-13. This is association evidence and does not prove effective supplementation.
Asthma/allergic airway inflammation extrapolated evidence	Childhood asthma and lung function	VitA	(9)	Observational study	VitA may maintain airway epithelial integrity and immune homeostasis.	Dietary VitA intake was associated with lung function and incident asthma in childhood. This may support a shared-airway inflammation context but should not be treated as direct AR evidence.
	ILC2/ILC3-mediated type 2 barrier inflammation	VitA/RA	(21–23)	Basic/translational mechanistic study	RA suppresses ILC2 proliferation and IL-5/IL-13 secretion through RARα and induces IL-10^+^ regulatory ILCs; VitA deficiency alters the ILC2/ILC3 balance.	Supports the mechanistic plausibility of RA-mediated regulation of type 2 mucosal inflammation. For AR, this should be considered mechanistic extrapolation or nasal-inflammation-related mechanism rather than AR clinical efficacy evidence.
	OVA-induced asthmatic mice	VitC	(40)	Animal experiment	High-dose VitC increases the Th1/Th2 cytokine ratio and reduces eosinophil infiltration.	Attenuates allergic lower-airway inflammation. This supports only mechanistic extrapolation for VitC and does not prove improvement of AR symptoms.
	Mouse model of allergic airway disease	VitC combined with choline chloride and selenium	(41)	Animal experiment/combined intervention	The combined intervention suppresses NF-κB, reduces oxidative stress, enhances IL-10 and Foxp3^+^ Tregs, and lowers IL-4/IL-5 and specific IgE/IgG1.	Reduces airway hyperresponsiveness and inflammatory-cell infiltration. Because this is a combined intervention, the effect cannot be attributed to VitC alone; for AR, it provides only indirect mechanistic support.
	Maternal VitD and childhood asthma, wheeze, and atopy	VitD	(54–56)	Meta-analysis/birth cohort	Fetal VitD status may affect immune development and maternal inflammatory status, including CRP-related pathways.	Low maternal VitD was associated with increased risk of childhood asthma, wheeze, or atopy. For AR, this is background evidence within the allergic-disease spectrum and should not be presented as direct AR-prevention evidence.
	ILC2 plasticity in human nasal inflammation	VitD	(62)	Basic/translational mechanistic study	1,25(OH)₂D₃ suppresses RORγt, blocks the conversion of ILC2s into IL-17-producing cells, and reduces IL-9.	Regulates ILC2 plasticity in nasal inflammation. This evidence is close to nasal AR mechanisms, but it is still not complete AR clinical evidence.
	Mouse asthma model	VitE	(82)	Animal experiment	Severe VitE deficiency enhances IL-5, TNF-α, ICAM-1, and IgE expression.	Aggravates OVA-induced allergic airway inflammation. This supports the possibility that VitE deficiency may amplify type 2 inflammation, but it remains asthma-extrapolated evidence.
	Allergic airway inflammation/ILC2s	VitE/α-T	(83)	Animal/mechanistic study	α-Tocopherol induces ILC2 ferroptosis through a SCARB1–LKB1-related pathway and decreases IL-5 and IL-13 secretion.	Alleviates allergic airway inflammation. This may support a possible mechanism for ILC2 regulation in AR, but validation in AR nasal mucosa is still required.
Mixed evidence: AR animal model + asthma extrapolation	Experimental AR and asthma united-airway model	VitE/γ-T	(86, 87)	Animal experiment/united-airway model	γ-Tocopherol scavenges RNS, inhibits COX/LOX, and reduces LTB4, IL-5, IL-13, and eosinophil infiltration.	Reduces inflammation and mucous-cell metaplasia in the nasal cavity, sinuses, lungs, and nasolacrimal ducts. The model directly involves experimental AR, but the evidence remains animal-based and has limited clinical translation.
Other allergic diseases/non-AR mechanistic evidence	Atopy; sex difference after neonatal supplementation	VitA	(12)	Clinical observation/cohort study	Early VitA exposure may affect immune maturation, and the direction of effect may be modified by sex.	Neonatal VitA supplementation was associated with increased atopy risk in girls. This highlights that VitA supplementation is not always protective and should not be used as evidence for AR treatment.
	*Alternaria alternata* mold allergy	VitA/RA	(24)	Allergen-specific mechanism/animal study	RA binds Alt a 1, masks IgE B-cell epitopes, reduces IgE binding and mast-cell degranulation, and enhances IL-10, TGF-β, and IgA.	Alleviates Alternaria allergic responses. This provides indirect mechanistic support for allergen-specific immune regulation, not clinical treatment evidence for AR.
	Delayed-type allergy/dendritic-cell regulation	VitA/9cRA	(20)	Basic/animal mechanistic study	9cRA induces OPN^+^ regulatory DCs and promotes Treg formation.	Enhances immune tolerance. This supports the basic mechanism of the VitA-regulated DC–Treg axis but is not AR clinical evidence.
	Atopic dermatitis, eczema, wheeze, and other offspring atopic risks	B vitamins	(29, 30)	Birth cohort/observational study	B vitamins participate in one-carbon metabolism and DNA methylation and may affect fetal immune programming.	Maternal folate, B12, or other B-vitamin intake/status was associated with offspring atopic dermatitis, eczema, or wheeze. This is evidence from other allergic diseases and does not directly support B-vitamin treatment for AR.
	Macrophage inflammatory response	VitB3/niacin	(31)	Cell-based mechanistic study	Niacin suppresses IL-1β, TNF-α, and IL-6 secretion through GPR109A.	Shows anti-inflammatory potential. The manuscript uses it as background evidence for B-vitamin immune regulation, not as direct AR evidence.
	Dendritic-cell inflammatory response	VitB7/biotin	(32)	Cell-based mechanistic study	Biotin deficiency suppresses AMPK activation, promotes TNF-α, IL-1β, IL-23, IL-12p40, and IL-6 secretion, and drives Th1/Th17 polarization.	Suggests that B-vitamin status can influence the inflammatory phenotype of DCs. This is non-AR mechanistic evidence and does not support direct supplementation therapy for AR.
	T-cell immune balance	VitB6	(33)	Basic mechanistic study	VitB6 deficiency reduces T-cell proliferation and the CD4/CD8 ratio, shifts immunity toward Th2, decreases IL-2, and increases IL-4.	May explain a potential relationship between B6 and allergic immune skewing, but it is not AR clinical evidence.
	Overall allergy-related symptoms	VitC	(35)	Long-term observational study/subgroup analysis	High-dose intravenous VitC may relieve symptoms through antioxidant and anti-inflammatory effects.	Reported improvement in allergy-related symptoms, but the disease target was not AR-specific. This can only be used as background evidence for the anti-allergic potential of VitC.
	Childhood eczema, food allergy, asthma, AR, and other allergic outcomes	VitD	(57–59)	Meta-analysis/birth cohort/systematic review of RCTs	Effects of maternal or infant VitD may be modified by baseline status, timing, dose, and genetic susceptibility.	Some studies did not find stable preventive effects of maternal or infant VitD supplementation on offspring allergic diseases. This supports the conclusion that evidence for VitD prevention remains controversial.
	Mast-cell-related allergic mechanisms	VitE	(88)	Cell-based mechanistic study	VitE inhibits the PI3K/PKB/GSK pathway and mast-cell proliferation, with different potency among isoforms.	Explains isoform-related differences and mast-cell regulation by VitE; this is not AR clinical evidence.
	Inflammatory injury model	VitE/γ-T	(89)	Animal/basic mechanistic study	γ-Tocopherol inhibits COX-2 and 5-LOX and reduces LTB4, TNF-α, 8-isoprostane, and LDH.	In this model, γ-tocopherol showed stronger anti-inflammatory effects than α-tocopherol. This should be limited to the specific model and not generalized to all AR contexts.

AR, allergic rhinitis; Breg, regulatory B cell; COX, cyclooxygenase; CRP, C-reactive protein; DCs, dendritic cells; IgE, immunoglobulin E; IL, interleukin; ILC2/ILC3, group 2/3 innate lymphoid cell; LTB4, leukotriene B4; NF-κB, nuclear factor-κB; OPN, osteopontin; OVA, ovalbumin; RA, retinoic acid; RCT, randomized controlled trial; RNS, reactive nitrogen species; ROS, reactive oxygen species; SAR, seasonal allergic rhinitis; SLIT, sublingual immunotherapy; TNF-α, tumor necrosis factor-α; TNSS, Total Nasal Symptom Score; Tregs, regulatory T cells; VDR, vitamin D receptor; Vit, vitamin; α-T, α-tocopherol; γ-T, γ-tocopherol; 9cRA, 9-cis-retinoic acid; 1,25(OH)₂D₃, 1,25-dihydroxyvitamin D₃.

Beyond immune cells, mucosal epithelial barrier integrity and the gut microbiota may represent additional layers through which vitamins influence AR. VitA and VitD have been implicated in maintaining mucosal immune homeostasis, whereas VitC and VitE may protect epithelial structures by reducing oxidative stress. In particular, the gut microbiota-VitA axis suggests that vitamin effects may depend not only on dietary intake or serum concentrations but also on microbiota composition and metabolic capacity. However, few studies have integrated nutritional status, gut microbiota profiles, nasal epithelial barrier function, and AR clinical phenotypes within the same cohort. Because VitA and VitD are fat-soluble vitamins with potential accumulation risk, supplementation strategies should also be considered within established nutritional safety limits. According to the IOM 2011 Dietary Reference Intakes, the tolerable upper intake level for VitD ranges from 1,000 to 4,000 IU/day depending on age, with 4,000 IU/day generally applied to adults (90). However, AR-related VitD studies have used highly heterogeneous regimens, ranging from approximately 800 IU/day to 60,000 IU/week, and Guo et al. (48) used 1,600 IU/day while noting that previous AR studies included doses up to 50,000 IU/week. These wide dose ranges indicate that no AR-specific optimal VitD supplementation regimen has been established, and higher weekly doses should be regarded as deficiency-correction strategies under medical supervision rather than routine adjunctive use. For VitA, the IOM/DRI defines the adult tolerable upper intake level for preformed vitamin A as 3,000 μg/day, mainly based on hepatotoxicity and teratogenicity risks. Therefore, no universal AR-specific target serum concentration or routine high-dose supplementation strategy can currently be proposed for either VitA or VitD. Future research should therefore combine microbiome analysis, mucosal immune profiling, and clinical outcome assessment to clarify whether vitamin-based interventions act through systemic immunity, local nasal immunity, microbiota-mediated pathways, or their interaction. A key unresolved issue is whether circulating vitamin concentrations are adequate proxies for tissue-level vitamin activity. For example, serum levels may not reflect local conversion into active metabolites, receptor expression in nasal mucosa, or microbiota-dependent metabolic availability. Therefore, future studies should move beyond serum vitamin measurements and include tissue- or cell-specific readouts, such as nasal epithelial VDR expression, local retinoic acid metabolism, oxidative stress markers, and microbiota-derived metabolic signatures.

Beyond the classical genomic and signaling pathways outlined above, vitamin-regulated miRNAs may provide an additional post-transcriptional layer through which nutritional status influences immune remodeling in AR. The clearest evidence currently comes from VitD and VitA-related pathways. In B cells, 1,25(OH)₂D₃ forms a VDR/RXR transcriptional repressor complex that suppresses the miR-17 ~ 92 cluster, particularly miR-19a, thereby relieving repression of IL-10, restoring IL-10 secretion, promoting a tolerogenic immune profile, and improving the response to allergen-specific immunotherapy (74). In parallel, RA cooperates with TGF-*β* to induce miR-10a in Tregs; by targeting Bcl-6 and Ncor2, miR-10a limits Treg conversion toward Tfh-associated phenotypes and, in the presence of RA, also restrains Th17 differentiation (17). These findings suggest that vitamins may regulate AR not only by altering cytokine production, IgE responses, MC activation, Eos recruitment, or ILC2 function, but also by acting upstream of miRNA networks that shape B-cell regulatory activity, Treg stability, Tfh differentiation, and Th17 polarization. This interpretation is supported by broader evidence from allergic airway diseases showing that miRNAs are involved in immune-cell differentiation, activation, airway remodeling, epithelial dysfunction, oxidative stress, NF-κB signaling, apoptosis, epithelial–mesenchymal transition, and Th1/Th2 imbalance. Several miRNAs, including let-7 family members, miR-21, miR-145, miR-146a/b, miR-155, miR-223, miR-29, and miR-375, have been associated with allergic inflammation, although most of these data derive from asthma, lower-airway models, environmental-exposure studies, or peripheral immune cells rather than AR-specific vitamin interventions. Therefore, these miRNAs should currently be regarded as candidate regulatory nodes rather than validated vitamin-responsive biomarkers in AR. Future studies should prioritize AR-relevant samples, including nasal epithelial cells, nasal lavage fluid, inferior turbinate tissues, and nasal mucosal immune-cell subsets, and integrate serum vitamin levels, dietary intake, local miRNA profiling, immune-cell phenotyping, transcriptomic and epigenomic datasets, and clinical outcomes such as symptom scores, medication use, IgE levels, nasal eosinophil counts, and responsiveness to allergen-specific immunotherapy. Such approaches may clarify whether vitamin-regulated miRNAs help explain delayed or heterogeneous responses to vitamin supplementation, identify patients most likely to benefit from nutritional adjunctive therapy, and uncover new targets for personalized immune modulation in AR.

Notably, previous clinical trials assessing VitC, VitD, and VitE in AR have reported inconsistent results. Heterogeneity in study design, including dosage regimens, baseline vitamin status, age, sex, disease severity, and concurrent treatments, contributes largely to these conflicting observations. For VitD, positive effects appear more evident in deficient patients, pediatric populations, males, or when supplementation is used as an adjunct to standard therapy, whereas studies on maternal or infant supplementation for long-term prevention remain inconclusive. This discrepancy may reflect differences between prevention and treatment settings, exposure timing, baseline VitD status, and outcome definitions. For VitE, divergent findings may also be related to allergen type and exposure pattern: seasonal allergic rhinitis during pollen exposure may be more responsive to short-term antioxidant modulation, whereas perennial allergic rhinitis involves continuous allergen stimulation and may require different endpoints or intervention strategies. In addition, VitE studies often assess total intake or serum levels without distinguishing individual tocopherol isoforms, which may obscure isoform-specific effects. Future studies should measure baseline vitamin concentrations, implement stratified randomization, conduct subgroup analyses based on age, sex, and disease severity, and standardize background treatments to clarify the real effects of vitamins and identify responsive populations.

From a clinical perspective, vitamin-based interventions should be regarded as individualized adjunctive strategies rather than alternatives to established AR treatments, including antihistamines, intranasal corticosteroids, and allergen-specific immunotherapy. Among the vitamins reviewed, VitD currently has the strongest translational potential, particularly in patients with insufficient baseline levels, pediatric populations, and settings combined with conventional pharmacotherapy or SLIT. By contrast, VitA and VitE are supported mainly by mechanistic evidence and limited clinical signals, whereas evidence for B vitamins and VitC remains insufficient or inconsistent in AR-specific studies. Therefore, routine supplementation cannot yet be recommended for all patients with AR. Clinical application should be guided by baseline nutritional status, correction of confirmed deficiency, avoidance of excessive intake, and safety monitoring, especially for fat-soluble vitamins. Future trials should distinguish deficiency correction from pharmacological supplementation, stratify participants by vitamin status, age, sex, disease severity, AR phenotype, and concomitant therapy, and incorporate standardized outcomes such as TNSS, medication use, quality-of-life scores, and nasal mucosal or immunological biomarkers. This approach may help identify responsive subgroups, clarify the true additive value of vitamin supplementation, and prevent premature extrapolation from mechanistic findings to routine clinical practice.

To overcome current limitations at the immune cell level, future research should move beyond descriptions of single vitamins and focus on interactive regulatory networks of combined vitamins, using multifactorial designs to identify optimal combinations for targeted immune cells. Clinical studies need to address safety concerns and establish daily upper intake limits, especially for the fat-soluble VitA and VitD, which carry a risk of excessive accumulation (91–93). These considerations suggest that vitamin supplementation should not be evaluated using a one-size-fits-all model. A vitamin may show benefit primarily in deficient individuals, in specific inflammatory endotypes, or when combined with conventional therapy. Conversely, supplementation in individuals with sufficient baseline levels may produce little benefit or even increase the risk of excessive accumulation, particularly for fat-soluble vitamins. Further exploration of optimal doses and administration timings should be undertaken to determine suitable vitamin types and levels for different stages of AR. Factorial trials combining vitamins with standard treatments are needed to evaluate whether vitamin supplementation can reduce drug dosage or extend remission duration. Furthermore, long-term real-world safety monitoring should be strengthened to establish safe upper limits and target serum ranges for fat-soluble vitamins, particularly in children and women of childbearing age. Future clinical trials should also distinguish between correction of deficiency and pharmacological supplementation. These two strategies have different biological assumptions, safety profiles, and target populations. Correction of deficiency aims to restore physiological immune homeostasis, whereas high-dose supplementation attempts to actively modulate inflammatory pathways. Failure to distinguish these concepts may obscure true therapeutic signals and partly explain the inconsistent results across existing studies. Only by integrating immune cell mechanisms, mucosal and microbiota-related pathways, population stratification and high-quality clinical evidence can vitamins be developed into reliable and precise adjuvant strategies for AR.

## Conclusion

Existing evidence indicates that multiple vitamins may participate in immune regulation relevant to AR. VitA, VitD, and VitE exert relatively well-described anti-inflammatory, antioxidant, and mucosal barrier-related effects in experimental and mechanistic studies. However, their clinical value in AR remains incompletely established, and available findings should be interpreted with caution. For vitamins B and C, the evidence is particularly limited: most data are indirect, inconsistent, or derived from non-AR allergic disease models. Therefore, these vitamins should not currently be presented as proven preventive or therapeutic agents for AR. Their specific mechanisms of action and clinical efficacy remain uncertain and require further verification through well-powered AR-specific studies.

Vitamins show potential as individualized adjunctive strategies in AR, and their effects are closely related to supplementation timing, dosage, baseline nutritional status, disease phenotype, and concomitant treatment. Future studies should distinguish correction of deficiency from pharmacological supplementation, because these two approaches have different biological assumptions, safety profiles, and target populations. Limitations of current research include unclear differences in therapeutic responses across populations, insufficient exploration of synergistic mechanisms among vitamins, limited validation of vitamin use combined with conventional treatments, and inadequate AR-specific mucosal evidence. Future studies should conduct targeted large-sample, multicenter clinical trials to determine appropriate populations and optimal supplementation regimens; elucidate the molecular mechanisms by which vitamins regulate nasal mucosal immunity and inflammatory pathways; and investigate synergistic effects between different vitamins as well as between vitamins and standard therapies. Only after these questions are addressed can vitamin-based interventions be developed into reliable, evidence-based, and precise adjunctive strategies for AR management.
